# Simple immunosensor for ultrasensitive electrochemical determination of biomarker of the bone metabolism in human serum

**DOI:** 10.3389/fchem.2022.940795

**Published:** 2022-08-26

**Authors:** Qiang Chang, Jie Huang, Liming He, Fengna Xi

**Affiliations:** ^1^ Shanxi Bethune Hospital, Shanxi Academy of Medical Sciences, Tongji Shanxi Hospital, Third Hospital of Shanxi Medical University, Taiyuan, China; ^2^ Tongji Hospital, Tongji Medical College, Huazhong University of Science and Technology, Wuhan, China; ^3^ Department of Chemistry, Key Laboratory of Surface & Interface Science of Polymer Materials of Zhejiang Province, Zhejiang Sci-Tech University, Hangzhou, China

**Keywords:** immunosensor, electroanalysis, ultrasensitive detection, biomarker of bone metabolism, osteocalcin

## Abstract

Ultrasensitive and selective determination of biomarkers of the bone metabolism in serum is crucial for early screening, timely treatment, and monitoring of the curative effect of osteoporosis, which is a silent disease with serious health threats. Immunoassay with a simple sensing interface and ultrahigh sensitivity is highly desirable. Herein, a simple electrochemical immunosensor is demonstrated based on gold nanoparticles (AuNPs) electrodeposited on chitosan-reduced graphene oxide (CS-G) composite modified electrode, which can achieve sensitive determination of the important biomarker of bone metabolism, bone gamma-carboxyglutamate protein (BGP). To overcome the agglomeration of graphene and introduce a biocompatible matrix with functional amino groups, CS-G is prepared and modified on the supporting glassy carbon electrode (GCE). Then, AuNPs are electrodeposited on CS-G through their interaction between amine groups of CS. The immobilized AuNPs provide numerous binding sites to immobilize anti-BGP antibodies (Ab_BGP_). The specific recognition between BGP and Ab_BGP_ results in a reduction in the mass transfer of the electrochemical probe (Fe(CN)_6_^3-/4-^) in solution, leading to a reduced electrochemical signal. Based on this mechanism, fast and ultrasensitive electrochemical detection of BGP is achieved when the concentration of BGP ranges from 100 ag ml^−1^ to 10 μg mL^−1^ with a limit of detection (LOD) of 20 ag ml^−1^ (S/N = 3). The determination of BGP in human serum is also realized with high reliability.

## Introduction

Osteoporosis is a silent disease that has become a serious health problem after cardiovascular disease. Osteoporosis is a systemic bone disease characterized by decreased bone mass and damage to the microstructure of bone tissue, leading to increased bone fragility and susceptibility to fractures (Walker-Bone et al., 2002; Rachner et al., 2011; Cheatham et al., 2017). In addition, the incidence of osteoporosis increases significantly with age. It is well known that fractures are the most common manifestation of osteoporosis. For instance, an osteoporotic fracture occurs every 3 s in the world. About 50% of women and 20% of men will experience the first osteoporotic fracture after the age of 50 years, and 50% of these patients might have a second osteoporotic fracture. Fractures may affect daily activities in mild cases, and patients will lose their ability to move independently or lead to cardiovascular and cerebrovascular accidents in severe cases (Price and Thompson, 1995; Afsarimanesh et al., 2018a; Lima et al., 2019). Bone mineral density (BMD) is mainly used to diagnose and monitor the curative effect of osteoporosis. Until now, methods to measure BMD include dual-energy X-ray absorptiometry (DXA), quantitative computed tomography (QCT), peripheral DXA, and quantitative ultrasound (QUS). However, these methods cannot accurately and quantitatively assess bone quality and are susceptible to problems such as osteophytes and calcification. In addition, the optimal treatment stage is often missed when bone density is abnormal. Unlike the slowly changing parameters of BMD, abnormal situations in markers of the bone metabolism might be detectable only within a few weeks (Farley et al., 1981; Christenson, 1997; Ivaska et al., 2005; Afsarimanesh et al., 2016; Afsarimanesh et al., 2018b). Combining imaging data and level of bone metabolism markers can realize early screening, timely treatment, and monitoring of curative effects of osteoporosis. Therefore, rapid and sensitive detection of bone metabolism markers is of great significance.

Osteocalcin, also known as bone gamma-carboxyglutamate protein (BGP), is an important biomarker of the bone metabolism. BGP has a molecular weight of ∼5.8 kDa and consists of 49 amino acids. Its total amount accounts for 15–20% of the non-collagen protein in bone tissue. BGP is synthesized and secreted by osteoblasts and ∼50% of its content enters the blood circulation. The main physiological function of BGP is to maintain the normal mineralization rate of bone, inhibit the formation of abnormal hydroxyapatite crystals, and inhibit the mineralization rate of cartilage (Calvo et al., 1996; Ivaska et al., 2005). Thus, serum BGP level can reflect the activity state of osteoblasts. Generally, the faster the bone turnover rate, the higher the BGP value. For instance, primary osteoporosis is the high conversion type, so BGP is significantly elevated. On the contrary, senile osteoporosis is a low-conversion type, resulting in no obvious increase in BGP (Ingram et al., 1994; Gundberg et al., 2002). Therefore, changes in BGP can be used to identify the types of osteoporosis and provide important references for studying the pathogenesis of bone diseases. The development of a convenient analysis of BGP with convenient fabrication, high sensitivity, good reliability, and low cost is highly desirable.

Electrochemical techniques have shown great potential in biological and environmental analyses (Yan et al., 2021a; Zheng et al., 2021; Gong et al., 2022a; Ma et al., 2022a; Wang et al., 2022). The electrochemical sensing platforms have been proven to offer the advantages of simple instrumentation, convenient to use, free of tedious pretreatment, easy integration, and miniaturization compared to chromatography methods which need trained operators and costly equipment (Lu et al., 2021a; Lu et al., 2021b; Zhang Y. et al., 2022). Furthermore, matrix effects caused by colorful contaminants can be effectively avoided compared with colorimetric and spectrographic strategies (Lu et al., 2021c). The construction of modified electrodes with good biocompatibility, high electron transfer rate, and easy immobilization of recognitive ligands is crucial to improving the performance of electrochemical sensors (Lin et al., 2020; Ma et al., 2020; Yan et al., 2020; Yan et al., 2021b; Wang et al., 2021). Recently, the introduction of functional nanomaterials to improve detection sensitivity and stability has become an important strategy to fabricate electrochemical sensors (Ma et al., 2022b; Zhang M. et al., 2022). Reduced graphene oxide (G) is a carbon nanomaterial with sp^2^-hybridized carbon atoms tightly packed into a single-layer two-dimensional (2D) honeycomb lattice structure. Owing to excellent optical, electrical, and mechanical properties and high charge transport properties, graphene has shown great potential in the fields of sensors, energy storage, and drug delivery (Gong et al., 2022b; Zou et al., 2022). However, G is prone to agglomerate because of the strong π–π interaction between graphene sheets. Biofunctionalization of graphene is effective to improve its hydrophilicity and biocompatibility (Kang et al., 2009; Jirakunakorn et al., 2020). Chitosan (CS), the product from natural polysaccharide chitin obtained through the removal of part of the acetyl group, has the characteristics of easy degradation and good biocompatibility. Numerous amino groups in CS can be used to immobilize functional substances such as proteins or nanoparticles (Qiu et al., 2009; Zhang J. et al., 2022). However, direct modification of electrodes using CS suffers from high interfacial resistance as CS is a non-conductive material. When G and chitosan are combined, the intercalation of CS between graphene layers can prevent the agglomeration of G sheets. On the other hand, reduced graphene oxide with a large conjugated structure can improve the conductivity of chitosan materials. In addition, chitosan-reduced graphene oxide nanocomposites (CS-G) can also provide a biocompatible microenvironment for biomolecules, which effectively promotes the maintenance of their activity (Kang et al., 2009). Gold nanoparticles (AuNPs) have also been widely used in bioanalysis due to their excellent electron transport ability, easy preparation, good biocompatibility, and controllable surface characteristics (Chen et al., 2018; Sarfraz and Khan, 2021; Tai et al., 2022). Combining CS-G with AuNPs is expected to easily construct high-performance electrochemical biosensors for highly sensitive detection of BGP.

**FIGURE 1:**
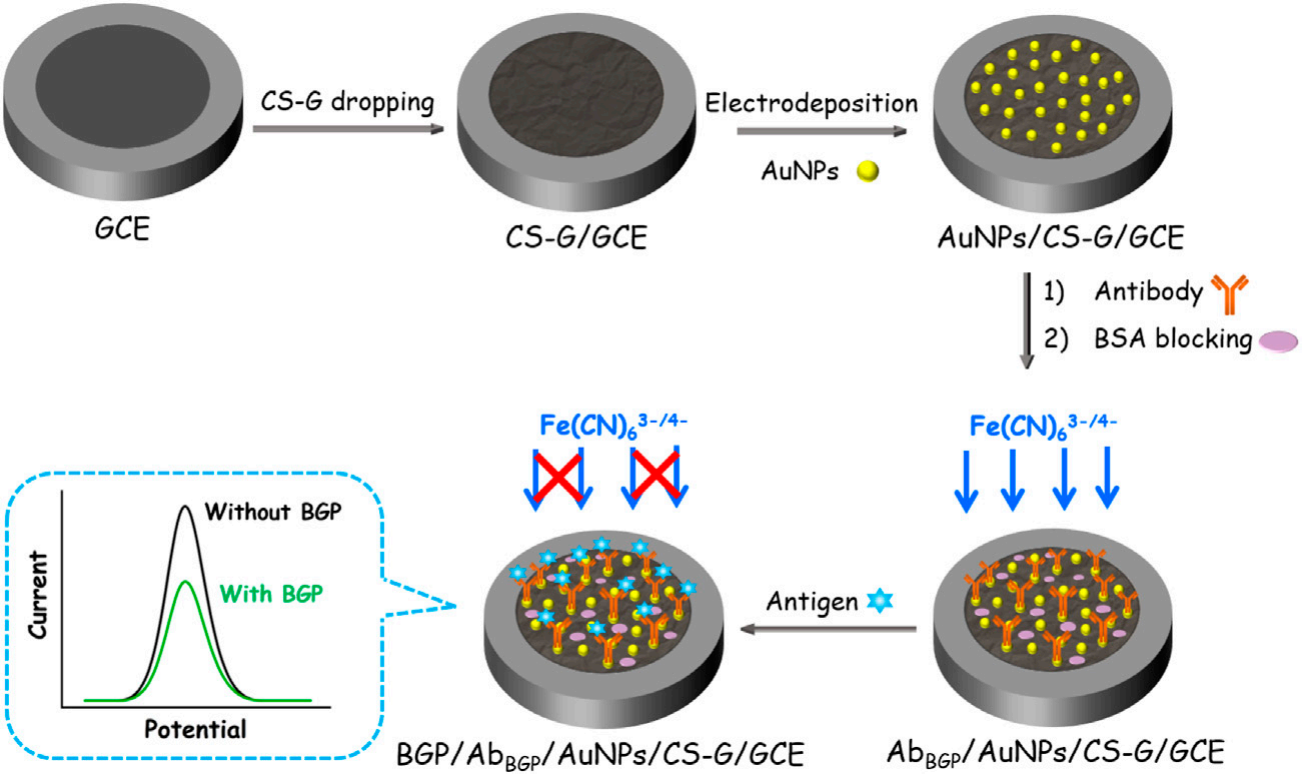
Schematic illustration for the simple fabrication of immunoanalysis interface and the following electrochemical detection of BGP.

In this work, we present a simple electrochemical immunoassay platform for sensitive detection of the important biomarker of the bone metabolism, bone gamma-carboxyglutamate protein (BGP), in human serum. As illustrated in Figure 1, chitosan–graphene nanocomposite (CS-G) is easily prepared and modified on the supporting glassy carbon electrode (GCE). Then, AuNPs are electrodeposited on CS-G through their interaction between amine groups of CS. The immunosensor (Ab_BGP_/AuNPs/CS-G/GCE) is finally obtained after anti-BGP antibodies (Ab_BGP_) are immobilized on AuNPs followed by the blocking of the non-specific sites with bovine serum albumin (BSA). The specific binding of BGP on the immunorecognitive interface results in a reduction in the mass transfer of the electrochemical probe (Fe(CN)_6_^3-/4-^) in solution, which leads to a reduced electrochemical signal. Based on this mechanism, fast and sensitive electrochemical detection of BGP is achieved. Combined with the advantages of simple fabrication, high sensitivity, good selectivity, and reproductivity, the immunosensor has great potential for sensitive and convenient detection of BGP in biological samples.

## Materials and methods

### Chemicals and materials

BGP antigen and anti-BGP antibody were purchased from Nanjing Okay Biotechnology Co., Ltd. (China). Prostate-specific antigen (PSA), carcinoembryonic antigen (CEA), carcinoma antigen 125 (CA125), and carcinoma antigen 199 (CA199) were purchased from Beijing KEY-BIO Biotech Co., Ltd. (China). S100 calcium-binding protein β was purchased from Proteintech (China). Potassium ferricyanide (K_3_[Fe(CN)_6_], 99.5%), potassium ferricyanide (K_4_[Fe(CN)_6_], 99.5%), bovine serum albumin (BSA), potassium chloride (KCl, analytical reagent-AR), chloroauric acid (HAuCl_4_·3H_2_O, 99.9%), sodium borohydride (NaBH_4_, 98%), sodium citrate (98%), and chitosan were purchased from Aladdin Biochemical Technology Co., Ltd. (China). Ethanol (99.8%) was purchased from Hangzhou Gaojing Fine Chemical Co., Ltd. (China). A glassy carbon electrode (GCE, 3 mm in diameter) was purchased from CHI instrument Co., Ltd. (China). Phosphate buffer solution (PBS) is prepared by Na_2_HPO_4_ and NaH_2_PO_4_. Ultrapure water (18.2 MΩ cm) used in the experiments is prepared by the Mill-Q system (Millipore Company).

### Measurements and instrumentations

The morphologies of G synthesized without the protection of CS, CS-G, and AuNPs/CS-G were investigated by scanning electron microscope (SEM, SU8010, Hitachi, Japan) with an acceleration voltage of 10 kV. The morphologies of GO and CS-G were investigated by transmission electron microscope (TEM, JEM-2100, JEOL, Japan) with an acceleration voltage of 200 kV. Energy dispersive X-ray spectroscopy (EDS) was performed on SU8010 SEM. The UV-Vis spectrum was measured using an ultraviolet spectrophotometer (UV-2450, Shimadzu, Japan). Fourier transform infrared spectroscopy (FT-IR) was measured using a Vertex 70 spectrometer (Bruker, United States) through the KBr tablet method. X-ray photoelectron spectroscopy (XPS) analysis was carried out on a PHI5300 electron spectrometer using 250 W, 14 kV, Mg Kα radiation (PE Ltd., United States). Raman spectra were measured using a 960FT-Raman spectrometer (Thermo Nicolet, United States). The XRD pattern was measured using a D8 Advance X-ray diffractometer (Bruker, Germany). Electrochemical impedance spectroscopy (EIS), cyclic voltammetry (CV), and differential pulse voltammetry (DPV) measurements were performed on an Autolab (PGSTAT302N) electrochemical workstation (Metrohm, Switzerland). All electrochemical measurements were performed at room temperature using a conventional three-electrode system. In brief, Ag/AgCl was used as the reference electrode. A platinum wire electrode was used as the counter electrode, and bare or modified GCE was used as the working electrode. The scanning rate for CV scanning was 50 mV/s. The parameters for DPV measurements included step potential (0.005 V), pulse amplitude (0.05 V), pulse time (0.05 s), and interval time (0.2 s).

### Synthesis of CS-G

Graphene oxide (GO) was prepared from natural graphite by a modified Hummers method (Santhiago et al., 2015). To prepare chitosan-modified composites (CS-G), GO dispersion (4 ml, 1 mg ml^−1^) was mixed with an aqueous solution of CS (36 ml, 0.25%, wt%, pH = 3) (Liu et al., 2012). A homogeneous dispersion was obtained by sonicating for 30 min. Then, hydrazine hydrate (50 wt%, 20 ml) was added to the above dispersion under rapid stirring and reacted in a water bath at 80°C for 3 h. The solid was collected by centrifugation at 15,000 rpm followed by washing three times with 0.1 mM HCl solution to remove the remaining CS. The CS-G was subsequently obtained and re-dispersed.

### Synthesis of AuNPs

Gold nanoparticles (AuNPs) were prepared by the electrodeposition method (Wu et al., 2015). A typical three-electrode system was adopted including a modified GCE as the working electrode, an Ag/AgCl electrode (saturated KCl) as the reference electrode, and a platinum sheet electrode as the counter electrode. In brief, the modified GCE was immersed in 0.5% HAuCl_4_, and a constant potential of −0.5 V for 2 s was applied. The electrode was then rinsed with ultrapure water.

### Fabrication of the immunosensor

GCE is used as the supporting electrode for the construction of the immunosensors. Before use, GCE was sequentially polished with 0.3 and 0.05 μm alumina slurry, and then ultrasonically cleaned in ethanol and ultrapure water for 60 s, respectively. The polished GCE has a glossy mirror under natural light. Then, 10 μL CS-G (0.25 mg ml^−1^) was drop-coated on the polished GCE. The obtained electrode was dried at 60 °C and denoted as CS-G/GCE. To electrodeposit AuNPs, CS-G/GCE was further immersed in 0.5% HAuCl_4_ and a constant potential of −0.5 V (vs. Ag/AgCl) for 2 s was applied. The electrode was then rinsed with ultrapure water to obtain AuNPs/CS-G/GCE. To fabricate the immunorecognitive interface, the BGP antibody (40 μL, 100 μg ml^−1^) was drop-coated on the surface of AuNPs/CS-G/GCE. After incubation at 37°C for 60 min, the electrode surface was rinsed with PBS (0.1 M, pH = 7.4) to remove unbound antibodies. The obtained electrode was then incubated with BSA solution (1%, wt%) for 60 min to block the non-specific sites followed by rinsing with PBS (0.1 M, pH = 7.4). The as-prepared immunosensor was denoted as Ab_BGP_/AuNPs/CS-G/GCE.

### Electrochemical determination of BGP

The Ab_BGP_/AuNPs/CS-G/GCE immunosensor was incubated with different concentrations of BGP (antigen) at 37°C for 40 min. KCl (0.1 M) containing Fe(CN)_6_^3-/4-^ (2.5 mM) was applied as the electrolyte. The electrochemical signal of the Fe(CN)_6_^3-/4-^ in the electrolyte before and after BGP binding was measured. For the real sample analysis, BGP in human serum (healthy male, provided by Shanxi Bethune Hospital, China) was determined using the standard addition method. To simulate the different BGP concentrations of osteoporosis patients, artificial BGP was added to the serum. Then, serum with added BGP was diluted by a factor of 50 with electrolyte and determined using the developed immunosensor.

## Results and discussion

### Easy fabrication of the immunosensor

Figure 1 illustrates the fabrication of the immunosensing interface. As illustrated, chitosan–graphene nanocomposite (CS-G) is prepared and modified on a glassy carbon electrode (GCE). The nanocomposite could overcome the agglomeration of reduced graphene oxide and introduce a biocompatible matrix with functional amino groups. Then, AuNPs are electrodeposited on CS-G, and the interaction between amine groups of CS and AuNPs/CS-G/GCE is obtained. Electrochemical synthesis of AuNPs has received much interest due to its controllable and green procedure. It is a simple, rapid, and convenient technique that can produce AuNPs with controlled characteristics (e.g., particle size, crystallographic orientation, mass, thickness, and morphology) by simply adjusting the electrodeposition parameters (Mohanty, 2010). The time-saving and environment-friendly electrodeposition process overcomes the drawbacks of chemical synthesis including the use of extra reagents, contamination from precursor molecules, and unwished by-products (Shein et al., 2009; Shedbalkar et al., 2014). Furthermore, more firm AuNP adherence to substrates can be realized by the electrodeposition method and facilitates the construction of the ultimate electrochemical device (Tai et al., 2022). The electrodeposited AuNPs provide numerous binding sites to immobilize anti-BGP antibodies (Ab_BGP_). After blocking the non-specific sites with bovine serum albumin (BSA), the immunosensor, denoted as Ab_BGP_/AuNPs/CS-G/GCE, is finally obtained. For the determination of BGP, the commonly used electrochemical probe, Fe(CN)_6_^3-/4-^, is applied as the solution-based redox indicator. When BGP specifically interacts with Ab_BGP_ on the surface of the electrode, the formed antigen–antibody complex hinders the mass transfer of Fe(CN)_6_^3-/4-^ in solution, leading to a significantly reduced electrochemical signal. Based on this mechanism, fast and sensitive electrochemical detection of BGP is achieved.

### Characterization of CS-G composite and AuNPs/CS-G-modified electrode

**FIGURE 2:**
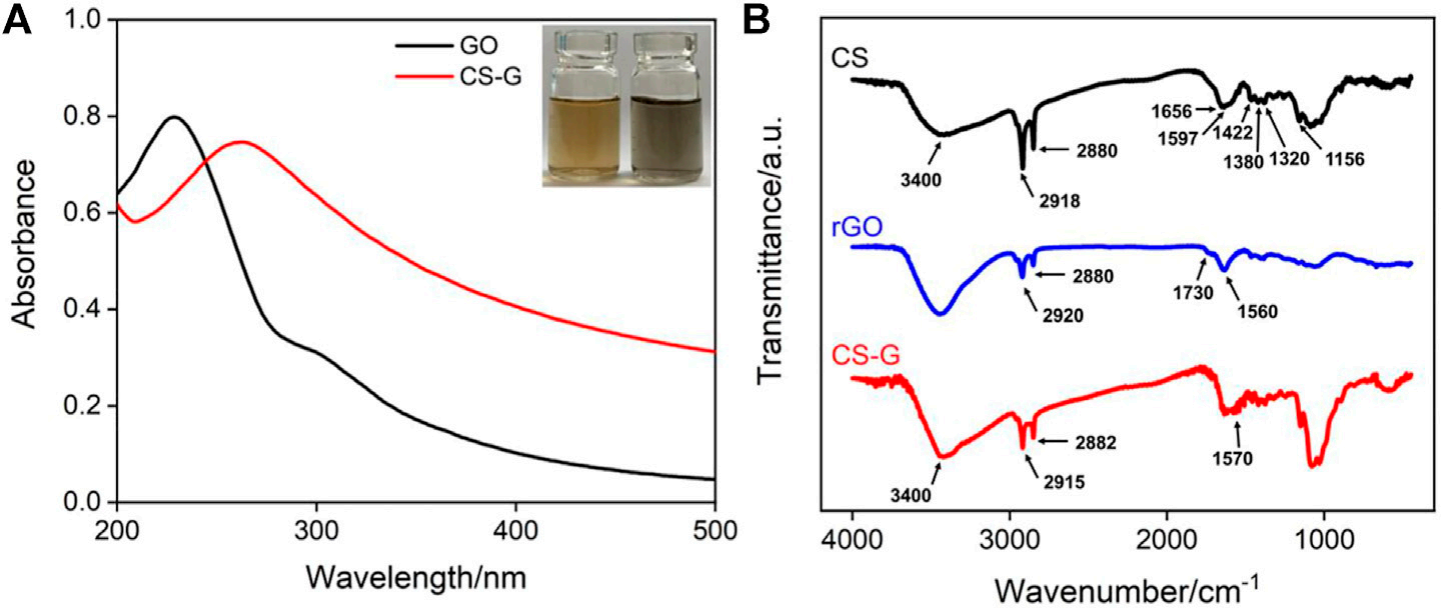
**(A)** UV-Vis absorption spectrum of GO and CS-G. Insets are photographs of GO (left) and CS-G (right) solutions. **(B)** FT-IR spectra of CS, rGO, and CS-G. The rGO was synthesized by reduction of GO using the same procesure but without CS.

The structure and morphology of CS-G are characterized by ultraviolet-visible spectroscopy (UV-Vis), Fourier transform infrared spectroscopy (FT-IR), and scanning electron microscopy (SEM). As shown in the inset of Figure 2A, the GO dispersion is a brown solution, while the CS-G dispersion is a black solution. The doping amount of chitosan on the CS-G nanocomposite is investigated by changing the mass ratio between the original GO and CS. Three ratios between CS and GO (5.62, 11.2, and 22.5) are employed to synthesize the CS-G nanocomposite. However, the synthesized dispersion can produce a large amount of precipitation after standing for 2 h at the low GO/CS ratio (5.62 and 11.2), which is attributed to the agglomeration of G when the protective agent CS is less. On the contrary, the high ratio between the used CS and GO (22.5) leads to stable dispersion of CS-G nanocomposite without precipitation. Thus, this doping amount of chitosan is chosen for further investigation. Figure 2A shows the UV-Vis absorption spectra of GO and CS-G. It can be seen that GO has two characteristic absorption peaks at 230 and 300 nm, corresponding to the π–π* transition of conjugated C-C=C and the n-π* transition of C=O, respectively (Ang et al., 2009; Guo et al., 2010). After being composited with CS, the absorption peak at 230 nm is red-shifted to 268 nm, indicating that GO is reduced and the electronic conjugation within the graphene sheets is restored upon hydrazine hydrate reduction (Li et al., 2008). In addition, the absorption peak at 300 nm disappears, further indicating the reduction of GO by hydrazine and the restoration of the conjugated carbon structure (Liu et al., 2012). These results prove the successful preparation of reduced graphene oxide. The changes in chemical composition during the preparation of CS-G are further characterized by FT-IR. As shown in Figure 2B, the characteristic peaks of hydroxyl and amino (3,400 cm^−1^), amide carbonyl (1,656 cm^−1^), N-H (1,597 cm^−1^), C-N (1,320 cm^−1^), and glycosidic bonds (1,156 cm^−1^) appear in the FT-IR spectrum of CS. In addition, the spectrum also reveals the characteristic absorption of saturated C-H (2,918 cm^−1^, 2,880 cm^−1^, 1,422 cm^−1^, 1,380 cm^−1^) (Mauricio-Sánchez et al., 2018). In the case of reduced graphene oxide (rGO), that was synthesized in absence of CS, the absorption peak (1,560 cm^−1^) attributed to the conjugated C-C=C framework significantly increases, indicating that GO was successfully reduced (Zhou et al., 2022). However, rGO still has a weak O=C-OH absorption peak (1730 cm^−1^) because the carboxyl group is difficult to be reduced. CS-G shows the absorption peak basically consistent with CS, indicating that CS does not change during the composite process (Li et al., 2008; Mianehrow et al., 2016). At the same time, the absorption peak of C-C=C proves the successful composite between CS and graphene.

X-ray photoelectron spectroscopy (XPS) is employed to analyze the structure and composition of GO and CS-G. As revealed by Supplementary Figures S1A,B (in supporting information-SI), the high-resolution C1s spectrums of GO and CS-G show four types of carbon atoms including C-C/C=C (284.6 eV, sp^2^ C), C-O (286.6 eV, epoxy and alkoxy), C=O (287.8 eV), and O-C=O (289.1 eV). However, the peak associated with C-/C=C becomes more dominant in CS-G, and the peaks related to the oxygen-containing carbon bonds especially C-O distinctly decrease, indicating the good reduction of GO (Zhou et al., 2022). Meanwhile, a new peak corresponding to the C-N bond appears at 286.0 eV in the spectrum of CS-G (Su et al., 2009), which is ascribed to the intercalation of chitosan. The N1s spectrum of CS-G shows two peaks at 399.9 and 401.9 eV, attributing to the N atom in -NH_2_ and/or -NH- groups and protonated species (-NH_3_^+^) of CS, respectively (Supplementary Figure S1C) (Jurado-López et al., 2017). These results confirm the successful synthesis of the CS-G nanocomposite.

Raman spectroscopy is one of the most powerful techniques to characterize the structural and electronic properties of graphene and its derivates (Zhu et al., 2010). There are usually two main features in the Raman spectrum of graphene, including the G band arising from the first order scattering of the *E*_2g_ phonon of sp^2^ C atoms (usually observed at ∼1,575 cm^−1^) and the D band arising from a breathing mode of κ-point photons of *A*_1g_ symmetry (∼1,350 cm ^−1^) (Guo et al., 2010). As shown in Supplementary Figure S2, the G band and D band are observed in spectra of both CS-G and GO. The relative intensity of the D band and G band is proportional to the average size of the sp^2^ domains (Guo et al., 2010), which increase from 0.87 of GO to 1.5 of CS-G, indicating the successful reduction of GO and the synthesis of CS-G.

The crystal structures of GO and CS-G are investigated by XRD. Supplementary Figure S3 shows the XRD patterns of CS-G and GO. As shown, GO has a feature diffraction peak at 2*θ* = 10.2° (*001*) with an interlayer *d*_001_ spacing of 0.864 nm (Patil et al., 2009; Zhu et al., 2010). For CS-G, the peak located at 10.2° becomes significantly weaker, confirming the great reduction of GO. A new peak located at 22° appears in the case of CS-G, related to the backbone of CS, suggesting the successful synthesis of CS-G (Mianehrow et al., 2016). Transmission electron microscope (TEM) images of GO and CS-G are displayed in Supplementary Figure S4. As shown, GO exhibits a thin stacked lamellar structure with some wrinkles (Rathnayake et al., 2017). After being reduced by hydrazine hydrate and combined with CS, the characteristic wrinkled sheet structure is retained, and some shadows corresponding to CS can be observed, which illustrates the successful synthesis of CS-G.

**FIGURE 3:**
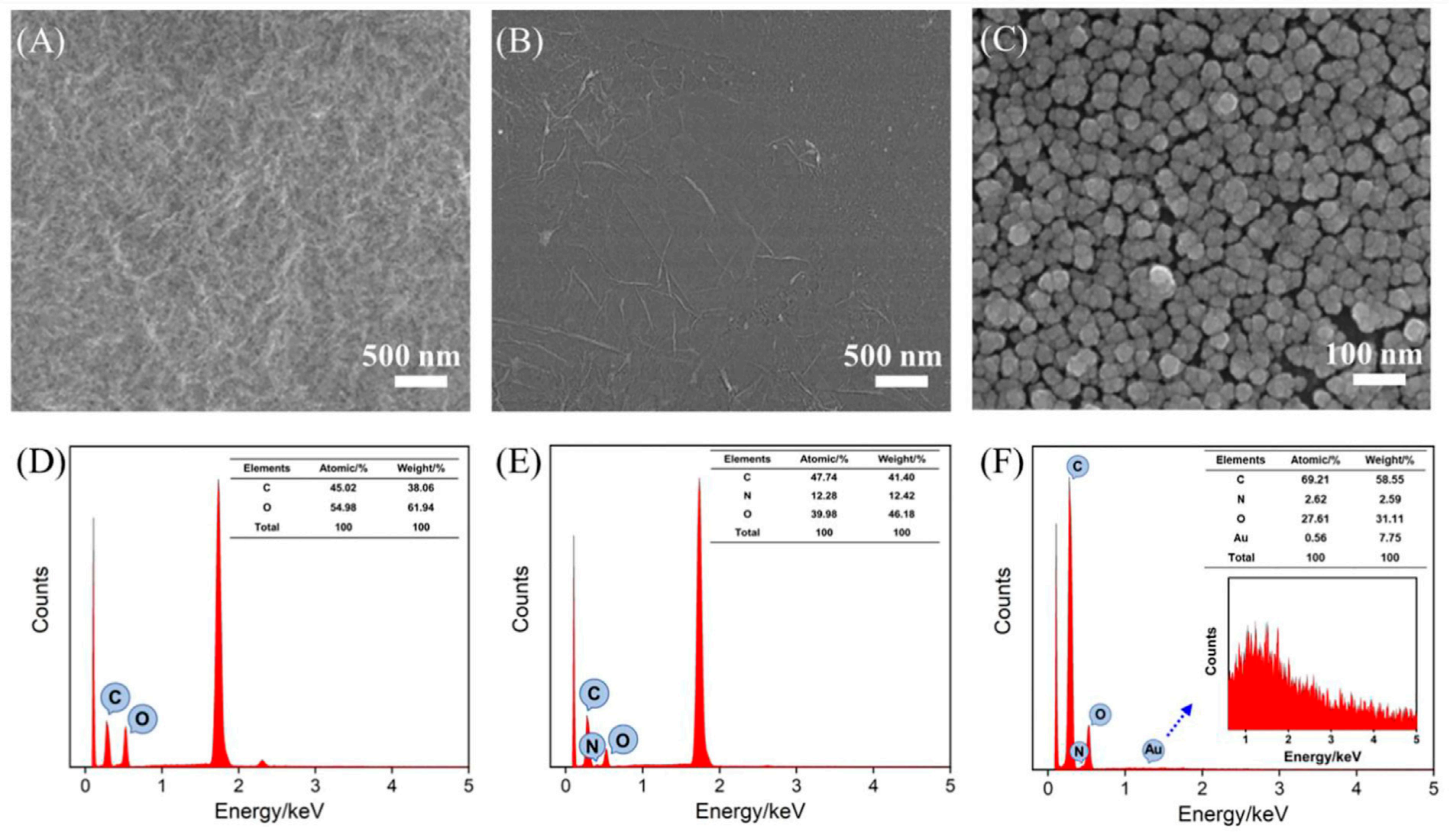
SEM image of G synthesized without the protection of CS **(A)**, CS-G **(B)**, and AuNPs/CS-G **(C)** modified glassy carbon sheet. EDS of GO **(D)**, CS-G **(E)**, and AuNPs/CS-G **(F)**. Inset in **(F)** is the magnified EDS of the Au section.

The morphologies of G synthesized without CS, CS-G, and the following AuNPs/CS-G modified electrode have been characterized by SEM and energy dispersive spectroscopy (EDS). A glassy carbon sheet is used to simulate the surface of GCE. As revealed in Figures 3A rGO that is reduced from GO by hydrazine without the protection of CS exhibits crumpled and aggregated structure owing to the possible aggregation (Abdolhosseinzadeh et al., 2015; Maddumage et al., 2022). When G is reduced in the presence of CS, the obtained CS-G exhibits a characteristic structure with the wrinkled sheet (Figure 3B). In addition, AuNPs with an approximate diameter of 40 nm uniformly distributed on the CS-G surface (Figure 3C). Energy dispersive spectroscopy (EDS) result further suggests the successful synthesis of CS-G and assembling of AuNPs (Figures 3D–F). The optimization of the amount of CS-G modification is studied by dropping 10 μL of CS-G with a different concentration on GCE. The peak currents obtained on the as-prepared CS-G/GCE in Fe(CN)_6_^3-/4-^ probe solution are compared. As shown in Supplementary Figure S5, the peak currents decrease as the amount of modified CS-G on the electrode increases, resulting from the poor conductivity of CS which may hinder the electron transfer. Thus, the optimal concentration of CS-G is set as 0.1 mg ml^−1^.

### Fabrication of immunosensor

**FIGURE 4:**
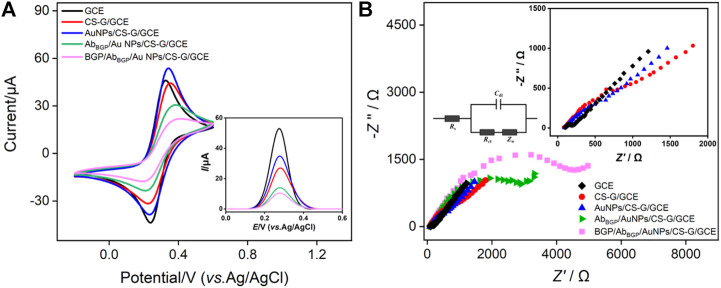
CV **(A)** and EIS **(B)** curves obtained on different electrodes including GCE, CS-G/GCE, AuNPs/CS-G/GCE, Ab_BGP_/AuNPs/CS-G/GCE, and BGP/Ab_BGP_/AuNPs/CS-G/GCE. The electrolyte solution is Fe(CN)_6_^3-/4-^ (2.5 mM) containing 0.1 M KCl. Inset in **(A)** includes the corresponding DPV curves. Insets in **(B)** are equivalent circuits of detection (left) and the enlarged view of the EIS curves at the high frequency region.

The feasibility of the construction of the immunosensor is verified by electrochemical monitoring of the changes in the electrode interface during the modification. Figure 4A shows the CV and the corresponding DPV (inset) curves obtained on different electrodes in Fe(CN)_6_^3-/4-^ solution. As shown, Fe(CN)_6_^3-/4-^ displays a pair of reversible redox peaks on GCE. When a layer of CS-G is modified on the surface of GCE, the peak current decreases, and the peak-to-peak difference increases. This is attributed to the poor electrical conductivity of CS. Further assembly of AuNPs could be achieved by forming Au-amine bonds between AuNPs and abundant -NH_2_ groups on CS-G. AuNPs/CS-G/GCE has a larger peak current and smaller peak-to-peak difference than CS-G/GCE due to the excellent electrical conductivity of AuNPs. The BGP antibody is then immobilized on AuNPs followed by blocking the non-specific site with BSA. BGP can bind on the as-prepared immunosensor through specific recognition between antigen and antibody. As seen, the peak current of the electrode decreases, and the peak-to-peak difference increases upon BGP binding. When BGP forms a non-conductive layer on the electrode surface, the electron transfer of the electrochemical probes on the electrode interface is hindered. These results demonstrate the successful construction of the immunosensor. According to CV curves obtained on GCE, CS-G/GCE, and AuNPs/CS-G/GCE (Figure 4A), the standard heterogeneous rate constant (*k*_s_) can be calculated by the following Nicholson equation (Nicholson, 1965):

$$\mathrm{\psi =}\frac{{\left(D_{0}/D_{R}\right)}^{\alpha /2}k_{s}}{{\left[D_{0}\pi v\left(nF/RT\right)\right]}^{1/2}},$$

where *D*_O_ and *D*_R_ are the diffusion coefficients of Fe(CN)_6_^3-^ and Fe(CN)_6_^4-^, respectively. *D*_O_ = *D*_R_ = 1 × 10^–5^ cm^2^ s^−1^ *v* is the scan rate of CV. *n* is the number of electrons transferred (*n* = 1). *F* is the Faraday constant (96,485 C mol^−1^). *R* is the gas constant (8.314 J mol^−1^ K^−1^). *T* is the absolute temperature (*T* = 298 K). The value of *ψ* can be obtained according to the peak-to-peak difference of CV. Thus, *k*_s_ of GCE, CS-G/GCE, and AuNPs/CS-G/GCE are calculated as 9.4 × 10^–3^ cm s^−1^, 4.3 × 10^–3^ cm s^−1^, and 4.7 × 10^–3^ cm s^−1^, respectively. As can be seen, after the modification of poor conductive CS-G, *k*_s_ of CS-G/GCE decreased obviously compared to GCE, which can be alleviated by the immobilization of AuNPs with good electrochemical properties.

Supplementary Figure S6A displays CV curves obtained on GCE or AuNPs/CS-G/GCE in PBS (0.1 M, pH = 5), where the non-Faraday current is proportional to the double layer capacitance (*C*_dl_) and can act as a quantitative indicator of the electrochemical active surface area (ECSA) of electrodes (Wei et al., 2019). As seen, a remarkably increased capacitive current (∼7 fold increasing) is observed on AuNPs/CS-G/GCE compared with that of GCE, suggesting an enlarged ECSA owing to the decoration of AuNPs. In addition, two apparent redox peaks at ∼1.1 and ∼0.55 V are observed on AuNPs/CS-G/GCE attributing to the decorating of AuNPs (Sierra-Rosales et al., 2018). At the same time, AuNPs/CS-G/GCE demonstrates larger decomposition currents and reduced decomposition potentials for both the anodic and cathodic limits, indicating an improved electroanalytical reactivity. The exact ECSA of GCE is calculated to be 0.06644 cm^2^ using reversible probe K_3_[Fe(CN)_6_] by the Randles-Sevcik equation (Supplementary Figure S6B) (Alam and Deen, 2020). The ECSA of AuNPs/CS-G/GCE is 0.465 cm^2^, indicating a highly increased active surface through the modification.

Electrochemical impedance spectroscopy (EIS) is also used to investigate the changes in the electrode interface during sensor construction. As shown in Figure 4B, each EIS curve consists of a semicircle in the high-frequency region and a linear part in the low-frequency region, where the former represents electron transfer-limited processes and the latter represents diffusion-limited processes. The left inset of Figure 4B illustrates the illustration of the equivalent circuit, which contains solution resistance (*R*_s_), double-layer capacitance (*C*_dl_), Warburg impedance (*Z*_w_), and apparent charge transfer resistance (*R*_ct_). The right inset is an enlarged view of the high-frequency region curves. The equivalent diameter of the semicircle in the high-frequency region is the apparent charge transfer resistance *R*_ct_. The *R*_ct_ of different electrodes is summarized in Supplementary Table S1. As seen, after modifying GCE with CS-G that has CS with poor conductivity, the *R*_ct_ of CS-G/GCE demonstrates a distinct increasement compared with that of GCE. Thanks to the excellent electrochemical property of AuNPs, the *R*_ct_ of AuNPs/CS-G/GCE decreases. After the combination of Ab_BGP_ and BGP, the *R*_ct_ further increases, indicating the successful construction of the immunosensor.

### Electrochemical determination of BGP

**FIGURE 5:**
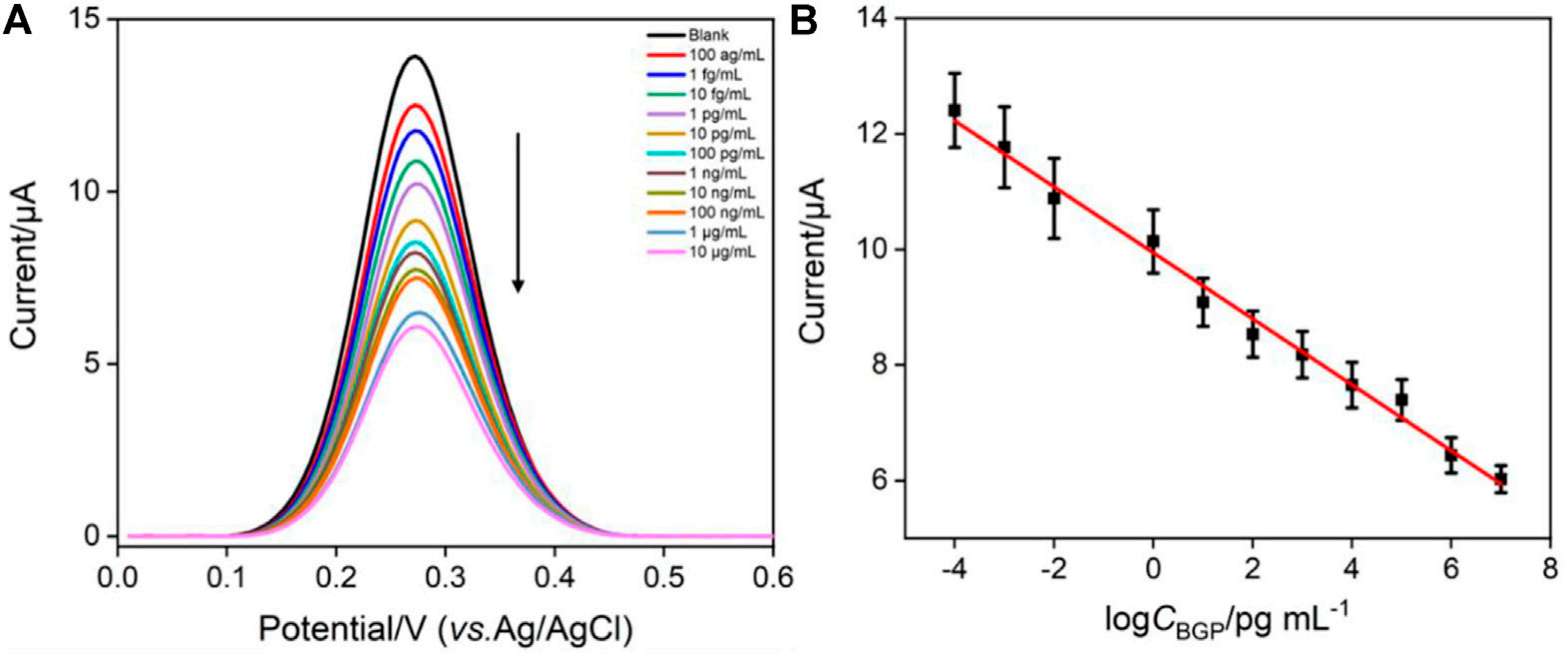
**(A)** DPV curves obtained on the developed immunosensors after incubation with different concentrations of BGP. **(B)** Corresponding linear regression curve. Error bars represent the standard deviation of three measurements.

Differential pulse voltammetry (DPV) is used to investigate the detection performance of the constructed immunosensor. Figure 5A presents the DPV curves obtained after incubating different concentrations of BGP on the immunosensors. As seen, the peak current decreases with increasing BGP concentration. This is attributed to the formation of the antigen–antibody complex through bio-specific recognition, which inhibits the electron transfer of the electrochemical probe on the electrode interface. This hindering effect becomes more obvious with the increase of bound antigen. When the concentration of BGP ranges from 100 ag ml^−1^ to10 μg mL^−1^, the peak current (*I*) of the electrode has a linear relationship with the logarithmic value of BGP concentration (log*C*_BGP_) (Figure 5B, *I* = -0.571 log*C*_BGP_ +9.94, *R* ^2^ = 0.990). The limit of detection (LOD) is 20 ag ml^−1^ (S/N = 3). A comparison between the determination of BGP using different methods is demonstrated in Supplementary Table S2 (Khashayar et al., 2017; Inal Kabala et al., 2019; Han et al., 2020; Bi et al., 2021). The LOD is lower than that obtained from the iron oxide material modified interdigitated electrode (IOM/IDE) (Bi et al., 2021), chemiluminescent immunoassay (Han et al., 2020), ethyl acetate/1,4-butanediol diglycidyl ether/6-mercaptohexanol modified gold electrode (EA/1,4-BED/6-MCH/AuE) (Inal Kabala et al., 2019), and AuNP-modified gold electrode (AuNPs/AuE) (Khashayar et al., 2017). The detection linear range is wider than that obtained using IOM/IDE, EA/1,4-BED/6-MCH/AuE, and AuNPs/AuE mentioned earlier. In comparison with other detection strategies (e.g., electrochemiluminescence), the electrochemical sensor has the advantages of simple instrumentation, easy operation, and the potential for the detection of colored or opaque samples (Zhang et al., 2020; Zhu et al., 2022).

### Selectivity, reproducibility, and stability of the constructed immunosensor

**FIGURE 6:**
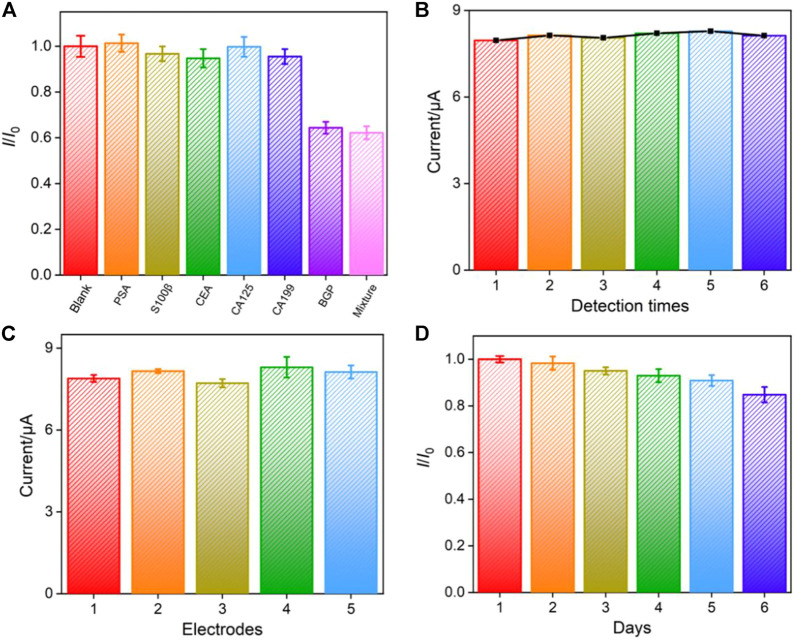
**(A)** Relative ratio of the current (*I/I*_0_) obtained on the developed immunosensors before (*I*_0_) and after (*I*) incubation with prostate-specific antigen (PSA, 1 ng ml^−1^), S100 calcium-binding protein β (S100β, 1 ng ml^−1^), carcinoembryonic antigen (CEA, 1 ng ml^−1^), carcinoma antigen 125 (CA125, 1 μU mL^−1^), carcinoma antigen 199 (CA199, 1 μU mL^−1^), BGP (1 ng ml^−1^), or the mixture of the above proteins. Repeatability **(B)**, inter-electrode reproducibility **(C)**, and storage stability **(D)** of the fabricated immunosensor. Error bars represent the standard deviation of three measurements.

To investigate the selectivity of the constructed immunosensor, the Ab_BGP_/AuNPs/CS-G/GCE is incubated with other tumor markers including prostate-specific antigen (PSA), carcinoma antigen 125 (CA125), S100 calcium-binding protein β (S-100β), cancer antigen 125 (CA125), and cancer antigen 199 (CA199). As shown in Figure 6A, the peak current of the electrode did not change significantly in the presence of one of these abovementioned proteins. Even if BGP is mixed with all of these tumor markers, the peak current of the electrode is not significantly different from that obtained with BGP alone. This result proves the specific recognition ability between antigen and antibody, indicating the excellent selectivity of the constructed immunosensor. The signal stability, inter-electrode reproductivity, and storage stability of the constructed immunosensor are also investigated. After the immunosensor is incubated with BGP, the electrochemical signal of the electrode was measured five consecutive times. A relative standard deviation (RSD) of the current value is 1.4% (Figure 6B). The reproducibility of the immunosensor electrodes was evaluated by preparing five electrodes in the same batch. The RSD for detecting BGP is 2.4% (Figure 6C). When the immunosensors are stored in a refrigerator at 4°C, the storage stability is studied using the peak measured on the first day and after storage, respectively. The immunosensor retains ∼90% of its original performance after 6 days of storage, indicating high stability.

### Determination of BGP in human serum

To evaluate the potential of the constructed immunosensor for practical application, the concentrations of BGP in human serum were determined by the standard addition method. Different concentrations of BGP are artificially added to the serum of a healthy man to simulate the different BGP concentrations of patients with osteoporosis. As shown in Supplementary Table S3, the immunosensor exhibits good recoveries ranging from 96.9 to 106.2% and low relative standard deviations (RSD <2.5%), suggesting good reliability and great potential in real sample analysis.

## Conclusion

In this article, an immunosensor is easily fabricated through a simple and convenient method, which can realize highly sensitive electrochemical detection of the biomarker of the bone metabolism, bone gamma-carboxyglutamate protein (BGP). The modification of the electrode with the chitosan–graphene nanocomposite (CS-G) increases the active area of the electrode and provides abundant amino sites to further anchor gold nanoparticles (AuNPs). On the one hand, AuNPs further improve the electron transfer at the electrode interface, and on the other hand, AuNPs could be used for the immobilization of recognitive antibodies. The specific binding of BGP to the recognitive antibody hinders the electron transfer of the electrochemical probe on the electrode surface, resulting in the reduction of the electrochemical signal. Based on this mechanism, a highly sensitive electrochemical detection of BGP is achieved when the concentration of BGP ranges from 100 ag ml^−1^ to 10 μg ml^−1^ with a limit of detection of 20 ag ml^−1^ (S/N = 3). The constructed immunosensor exhibits excellent selectivity, good reproducibility, and high stability. The determination of BGP in human serum is also achieved with high reliability. The simple construction and good performance of the developed immunosensor provide an efficient strategy for convenient and sensitive determination of bone metabolic markers.

## Data Availability

The original contributions presented in the study are included in the article/Supplementary Material; further inquiries can be directed to the corresponding author.
