# Supplementary material for: Simple immunosensor for ultrasensitive electrochemical determination of biomarker of the bone metabolism in human serum
Source: Front Chem. 2022 Aug 26;10:940795. doi: 10.3389/fchem.2022.940795 (PMC9458950; doi:10.3389/fchem.2022.940795)
Supplement: Supplementary file 1 [file DataSheet1.PDF]

TABLES

Table S1 R_{ct} of different electrodes.

Electrode	R_{ct}/Ω
GCE	137
CS-G/GCE	2501
AuNPs/CS-G/GCE	2004
Ab_{BGP}/AuNPs/CS-G/GCE	4750
BGP/Ab_{BGP}/AuNPs/CS-G/GCE	7597

Table S2 Comparison between determination of BGP using different methods.

Electrode materials	*Method*	*Detection range* (pg/mL)	*LOD* (pg/mL)	*Ref.*
AuNPs/CS-G/GCE	DPV	1×10^{-4}–1×10^{7}	2×10^{-5}	This work
IOM/IDE	LSV	10–3×10^{6}	100	63
—	CLIA	—	30	64
EA/1,4-BED/6-MCH/AuE	EIS	10–60	1.1	65
AuNPs/AuE	DPV	100–50000	100	66

AuNPs: gold nanoparticles; CS-G: chitosan-reduced graphene oxide; GCE: glassy carbon electrode; DPV: differential pulse voltammetry; LSV: linear sweep voltammetry; IOM: iron oxide material; IDE: interdigitated electrode; CLIA: chemiluminescent immunoassay; EA/1,4-BED/6-MCH/AuE: ethyl acetate/1,4-butanediol diglycidyl ether/6-mercaptohexanol modified gold electrode; AuE: gold electrode.

Table S3 Determination of BGP in serum samples.

Sample	Spiked (pg/mL)	Found (pg/mL)	RSD (%, n=3)	Recovery (%)
Serum[a]	0.100	0.0970	2.2	97.0
	10.0	10.6	2.5	106.2
	10000	9689.0	2.1	96.9

[a]Samples with added BGP were diluted by a factor of 50 using the electrolyte. The concentration of BGP was the added concentration before dilution.

FIGURES

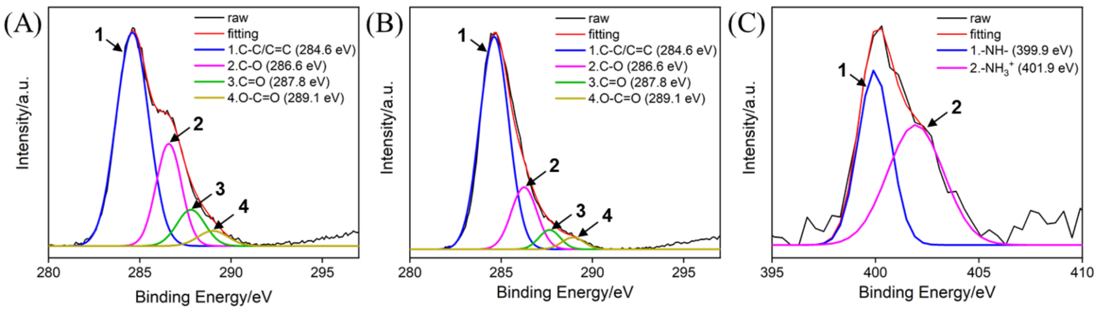

Figure S1 High-resolution C1s XPS profiles of GO (A) and CS-G (B). High-resolution N1s XPS profile of CS-G (C).

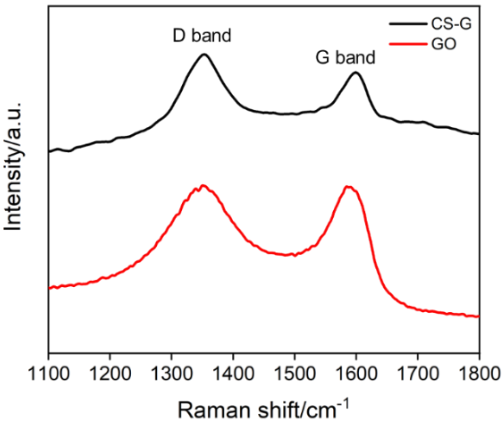

Figure S2 Raman spectra of CS-G and GO.

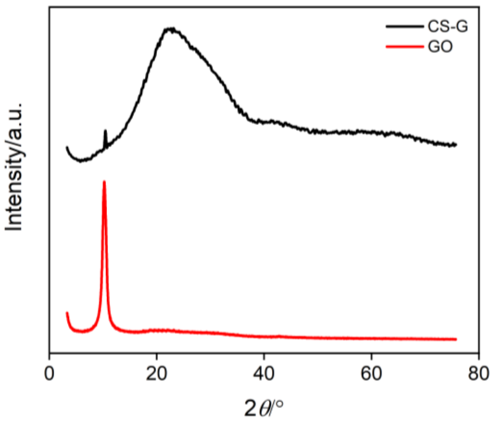

Figure S3 XRD patterns of GS-G and GO

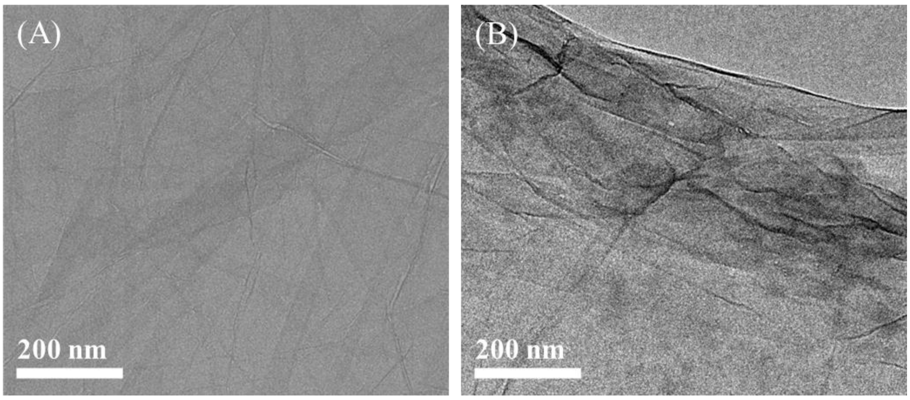

Figure S4 TEM images of GO (A) and CS-G (B).

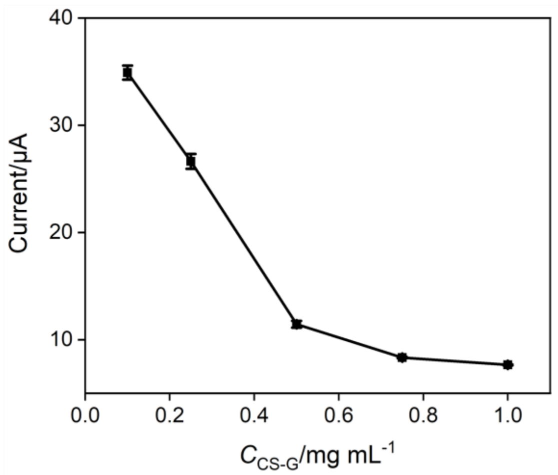

Figure S5 DPV peak current obtained on GCE modified with different amount of CS-G obtained in 0.1 M KCl containing 2.5 mM $Fe(CN)_6^{3-/4-}$.

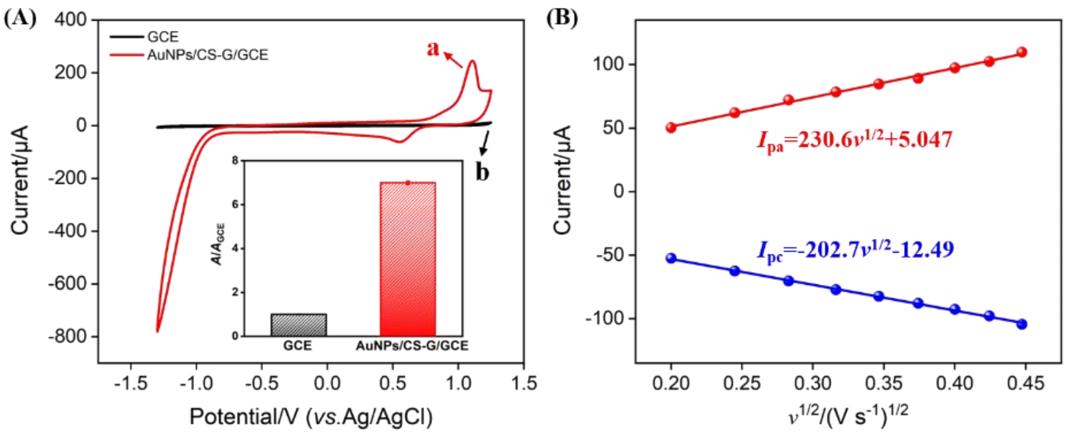

Figure S6 (A) CVs obtained on GCE or AuNPs/CS-G/GCE in 0.1 M PBS (pH=5) saturated with N_2. The scan rate is 0.1 V s^{-1}. (B) Relationship between I *vs.* $v^{1/2}$ derived from the CVs of GCE obtained in 5 mM $K_3[Fe(CN)_6]$.
